# Exploring the effects of resveratrol and β-hydroxy-β-methylbutyric acid under different protein levels on the ileal health of tibetan sheep

**DOI:** 10.3389/fmicb.2025.1612170

**Published:** 2025-07-21

**Authors:** Wei Gao, Kaina Zhu, Xianhua Zhang, Geerli Saren, Yu Zhang, Jiacheng Gan, Shengzhen Hou, Linsheng Gui

**Affiliations:** College of Agriculture and Animal Husbandry, Qinghai University, Xining, Qinghai, China

**Keywords:** resveratrol, β-hydroxy-β-methylbutyric acid, metabolomics, microbiota, Tibetan sheep

## Abstract

**Introduction:**

Resveratrol (RES) and β-hydroxy-β-methylbutyric acid (HMB) have been shown to improve growth performance in Tibetan sheep by regulating the gut microbiota. This study explored the effects of RES and HMB supplementation on the microbial composition and metabolite levels in the ileum of Tibetan sheep receiving diets with different protein levels.

**Methods:**

In a 2 × 2 factorial arrangement, consisting of dietary protein levels (12% and 14%) and feed additive levels (no addition; and RES 1.50 g/d and HMB 1.25 g/d addition). A total of 120 healthy two-month-old male Tibetan lambs (16.87 ± 0.31) were randomly divided into 4 groups (*n = 6*): 12% protein level group (L), the 12% protein level supplemented with RES and HMB group (L-RES-HMB); the 14% protein level group (H); and the 14% protein level supplemented with RES and HMB group (H-RES-HMB).

**Results:**

The results demonstrated that the activities of digestive enzymes (β-amylase, trypsin, lipase, and cellulase), the levels of antibodies (IgA and IgM), and the contents of short-chain fatty acid (SCFA) (butyric acid) were significantly increased in the H-RES-HMB group (*P* < 0.05). Additionally, RES and HMB supplementation affected the morphology of ileum tissue, improving the villus height, crypt depth, and mucosal thickness (*P* < 0.05). Microbial analysis revealed that compared with the L-RES-HMB group, the H-RES-HMB group had a higher abundance of *Planctomycetota*, *Solibacillus*, and *Paenibacillus* (*P* < 0.05). Metabolomics analysis revealed a total of 229 significantly different metabolites, of which Irinotecan, Erdosteine thioacid, 4,4’-diaminodiphenylmethane, and Morphine N-oxide emerged as the key up-regulated metabolites. These differential metabolites were mainly enriched in pathways such as protein digestion and absorption, metabolic pathways, and mineral absorption.

**Discussion:**

Overall, when the dietary protein content was 14%, digestive enzyme activities, immune responses, and SCFAs levels in the ileum were improved, and the mucosal morphology of the ileum was enhanced. When the 14% protein diet was supplemented with RES and HMB, the concentration of butyric acid was increased. This increase was due to the regulation of the ileum microbiota (Firmicutes and *Clostridium_sensu_stricto_1*) and metabolites (xanthine and uric acid), which promoted the activities of digestive enzymes and immune responses and improved mucosal morphology in the ileum.

## Introduction

In China, the Tibetan sheep is one of the most important rough-wool sheep breeds in the Tibetan Plateau region, with irreplaceable roles in the local ecology, economy and culture (Sha et al., 2022; Ma et al., 2023). As a key part of the digestive system in ruminants, the ileum is not only responsible for nutrient absorption, but also plays an important immune barrier function through immune cells and tight junction structures in the mucosal layer (Zhao et al., 2019). Studies have shown that the development and function of the ileum are influenced by a combination of dietary composition, microbial community and environmental factors (Wang Q. et al., 2021).

Resveratrol (RES), a natural stilbene and non-flavonoid polyphenol, holds significant medicinal value (Pasquariello et al., 2020). Research has shown that RES can protect the integrity of tight junctions in human Caco-2 colonic epithelial cells and enhance the function of the intestinal epithelial barrier. Additionally, RES is characterized by antioxidant, antibacterial, and anti-inflammatory properties and is known to influence metabolic regulation (Mayangsari and Suzuki, 2018b). Dietary RES supplementation can mitigate the adverse impacts of heat stress on intestinal morphology in broilers and promote the antioxidant capacity of the intestinal mucosa, thereby improving the growth of broilers under heat stress (Wang C. et al., 2021). Studies also demonstrate that RES can increase the digestibility of dry matter, neutral detergent fiber, acid detergent fiber, organic matter, and nitrogen in the diet (Chen et al., 2015). Studies in livestock indicate that specific dosages of RES modulate gastrointestinal microbiota: supplementation with 4 mg/kg body weight (BW) RES has been reported to rapidly increase the population of desulfurizing bacteria and reduce *methanogenic archaea* in Holstein calves (Zhang R. et al., 2019), while dietary inclusion of 0.25 g/d RES enhances the rumen abundance of *Fibrobacter succinogenes*, *Ruminococcus albus*, and *Butyrivibrio fibrisolvens* in sheep and inhibits the growth of protozoa and methanogens, thus regulating rumen microbiota composition (Ma et al., 2015). In rats, RES preserves intestinal barrier integrity and alleviates intestinal damage by inhibiting apoptosis of intestinal epithelial cells (Zhao et al., 2018). Currently, RES is utilized as a nutritional supplement because of its widespread benefits in preventing and managing various diseases (Meng et al., 2023).

β-hydroxy-β-methylbutyric acid (HMB), which is a metabolite originating from the essential amino acid leucine, has an anabolic effect. Studies have revealed that dietary HMB can improve the quality of poultry meat by promoting net protein synthesis and reducing skeletal muscle degradation (Kop Bozbay et al., 2024). Moreover, HMB can strengthen the immune system and prevent diseases in goat (Ząbek et al., 2016). HMB has been found to significantly reverse gut dysbiosis in mice on a high-fat diet, improving the diversity of the gut microbiota and the relative abundances of *Bacteroides* and fungi. Its effects are mediated by the reprograming of the gut microbiota and its metabolism, especially the production of propionic acid by *Bacteroides* (Duan et al., 2019).

Although studies have been conducted to investigate the effects of RES and HMB on ruminants individually, there is a lack of research on the synergistic effects of different protein levels with these two additives. The present study aimed to fill this gap by investigating the effects of adding RES and HMB to diets with different protein levels on digestive enzyme activities, immune indices, mucosal morphology and SCFAs content in the ileum of Tibetan sheep. This not only helps to gain a deeper understanding of the mechanism of action of RES and HMB in the ileum of ruminants, but also provides a scientific basis for optimizing the feeding management of Tibetan sheep.

## Materials and methods

The animal study was approved by animal care and experimental protocols were approved (QUA-2020-0710) by the Institutional Animal Care and Use Committee of the Qinghai University.

### Test animals and sample collection

The experiment took place at Kukunuoer Food Co., Ltd. in Haiyan County, Haibei Tibetan Autonomous Prefecture, Qinghai Province. 120 healthy 2-month-old male Tibetan lambs with an initial weight of 16.87 ± 0.31 kg were chosen for this research. A 2 × 2 design was adopted, and the lambs were randomly allocated to four parallel experimental groups, with 30 sheep in each group. The lambs were fed two different diets with different levels of crude protein (CP) (low: 12% and high: 14%) and feed additives (no addition and addition of 1.5 g/d RES and 1.25 g/d HMB). The experimental treatment groups were: 12% protein only (L), 12% protein with RES and HMB supplementation (L-RES-HMB), 14% protein only (H), and 14% protein with HMB and RES supplementation (H-RES-HMB). RES with a purity of > 99% was obtained from Xi’an Grass Plant Technology Co., Ltd. (Xi’an, China), whereas HMB of equivalent purity (> 99%) was procured from TSI Group Co., Ltd. (Shanghai, China). In the preparation of experimental feed, both RES and HMB were first incorporated into the premix before being directly combined with the concentrate components.

The composition and nutritional contents of the basal diet are presented in Table 1. The diet comprised concentrated feed, concentrated feed supplement, and roughage (oat hay and oat silage in a 1:1 ratio). The nutrient levels of feed ingredients were measured (Supplementary Table S1). A 7:3 concentrate-to-roughage mixing mode was adopted, and fresh feed was provided twice a day at 07:00 and 13:00. The fattening program was continued for 100 days (including 10 days for the pre-test and 90 days for the test period). At the end of the experiment, six Tibetan sheep with similar physical characteristics were selected from each group for slaughter in order to conduct subsequent analyses.

**TABLE 1:** Composition and nutrient levels of basal diet.

Items	L-CP	H-CP
**Ingredient (%)**		
Corn	58.30	51.50
Soybean meal	1.00	2.00
Rapeseed meal	7.00	12.80
Cottonseed meal	2.00	2.00
Palm meal	25.00	25.00
NaCl	1.00	1.00
Limestone	1.00	1.00
Baking soda	0.10	0.10
Premix^1^	4.60	4.60
Total	100.00	100.00
**Nutrient levels^2^**		
Digestible energy (MJ ⋅ kg^–1^)	12.84	12.71
Crude protein (%)	12.13	14.27
Ether extract (%)	3.44	3.29
Crude fiber (%)	11.05	11.64
Neutral detergent fiber (%)	26.04	26.70
Acid detergent fiber (%)	19.11	19.97
Ca (%)	0.80	0.84
P (%)	0.35	0.40

^1^Premixes provide Cu 18 mg, Fe 66 mg, Zn 30 mg, Mn 48 mg, Se 0.36 mg, I 0.6 mg, Co 0.24 mg, VA 24,000 IU, VD 4 800 IU, and VE 48 IU per kg of feed.

^2^Digestible energy is calculated and the rest is measured. (3) L-CP: The group with a crude protein level of 12%; H-CP: The group with a crude protein level of 14%.

### Sample collection and processing

Upon the completion of the experiment, all experimental sheep underwent a 12 h fasting period and a 2 h water deprivation before being slaughtered. The ileal contents were gathered into sterile and enzyme-free cryogenic tubes, promptly placed in dry ice, and then stored at −80°C for the subsequent DNA extraction, metabolite extraction, and 16S rDNA sequencing. Their gastrointestinal tracts were flushed with sterile PBS water, and the middle part of the ileum was excised and put into a sterile cryopreservation tube. The tissue was quickly frozen in liquid nitrogen and stored at −80°C for later analysis. Moreover, another 3 cm segment of ileal tissue was taken out and placed in a 4% paraformaldehyde fixative prior to sectioning for histological examination.

### Determination of ileal digestive enzyme activity

After thawing on ice, approximately 10 mL ileal contents were transferred to 15 mL centrifuge tubes and centrifuged at 3,000 rpm for 20 min at 4°C. The supernatant was collected, and the activities of ileal digestive enzymes including cellulase (YJ60100-96), trypsin (YJ60070-96), lipase (YJ60090-96), α-amylase (YJ60110-96), and chymotrypsin (YJ60080-96) were measured by an enzyme-linked immunosorbent assay (ELISA) from Enzyme Immuno Industry Co., Ltd. in Jiangsu, China. The specific experimental steps were carried out by the previous method described (Gan et al., 2024). A microplate reader (Rayto, RT-6100, Shenzhen Rayto Life and Analytical Sciences Co., Ltd., China) was employed for imaging at 450 nm.

### Determination of ileal immune indexes

After thawing, about 1 g of ileal tissue was homogenized in 1 mL PBS buffer solution and then centrifuged at 3,000 × g, 4°C for 20 min. The immune indexes of supernate, such as immunoglobulin A (IgA, YJ60010-96), immunoglobulin M (IgM, YJ60030-96), immunoglobulin G (IgG, YJ60020-96), tumor necrosis factor-α (TNF-α, YJ60040-96), and interleukin-1β (IL-1β, YJ60050-96) were measured using the ELISA (Rayto, RT-6100, Shenzhen Rayto Life and Analytical Sciences Co., Ltd., China) by the previous method described (Ji et al., 2024a).

### Examination of mucosal morphology in the ileum

The fixed ileal tissues (4% paraformaldehyde solution, 48 h) were embed in paraffin for sectioning. The prepared tissue sections were placed under microscope (OLYMPUS, DP26, Tokyo, Japan), and then 5 fields of vision with good trends for each section were selected randomly to measure villus width, villus height, crypt depth, muscular layer thickness, and mucosal thickness with the MShot Image Analysis System. Three sets of data were recorded in each field, and villus height/crypt depth (V/C) ratio was computed.

### Determination of the SCFAs content in the ileum

For the separation of SCFAs, an Agilent DB-FFAP capillary column (30 m × 250 μm × 0.25 μm) gas chromatography system was used. The concentrations of SCFAs were determined using a gas chromatography-mass spectrometry system (7890B GC System, Agilent, Billerica, MA, United States). Helium served as the carrier gas with a flow rate set at 1.2 mL/min. The injection process adopted the split mode, with a split ratio of 5:1 and an injection volume of 1 μL. The oven temperature was initially maintained at 50°C for 1 min, then increased to 220°C at a rate of 18°C per min and held at this temperature for 5 min. Each sample underwent analysis under the multiple reaction monitoring mode. Moreover, the temperatures of the injector inlet and transfer line were 250 and 230°C, respectively (Bianchi et al., 2011). The chromatographic peak area and retention time were extracted via MSD ChemStation software (G1701AA, Agilent Technologies, Inc., Santa Clara, CA, United States). Prepare standard solutions at concentrations of 0.005, 0.02, 0.05, 0.1, 0.2, 0.3, 0.5, 1, 2, 5, 8, 10, and 20 μg/mL. Obtain the chromatographic peak intensity data of the quantitative signals for each concentration. Plot the standard curves for different substances, with the ratio of external to internal standard concentrations as the x-axis and the ratio of external to internal standard peak areas as the y-axis. A standard curve was drawn to calculate the content of SCFAs in the ileum (Lotti et al., 2017).

### 16S rDNA sequencing analysis

The DNA of the ileal contents was extracted using the HiPure Fecal DNA Extraction Kit (Magen, Guangzhou, China) according to the kit instructions. The 16S rDNA V3-V4 region was amplified with primers 341F (5′-CCTACGGGGNGGCWGCAG-3′) and 806R (5′-GGACTACHVGGGGTATCTAAT-3′). The amplification conditions were 95°C for 5 min, followed by 30 cycles of 95°C for 1 min, 60°C for 1 min, 72°C for 1 min, and finally 72°C for 7 min. The Illumina DNA Prep Kit (Illumina, San Diego, CA, United States) was used to prepare the sequencing library. The raw data were uploaded to the NCBI Sequence Read Archive (SRA) database.

Low-quality reads were filtered using the FASTP software (version 0.18.0) (Chen et al., 2018). Then, the paired-end reads were assembled into tags by the FLASH software (version 1.2.11) (Magoc and Salzberg, 2011). Subsequently, the tags were filtered to obtain Clean tags (Bokulich et al., 2013). Based on the Clean tags, the UPARSE algorithm of the USEARCH software (version 9.2.64) was adopted for OTU clustering (Edgar, 2010). The UCHIME algorithm was utilized to remove the chimeric tags detected during the clustering alignment process, forming Effective tags (Edgar et al., 2011). Finally, the OTU abundance statistics were completed based on the Effective tags. The Alpha diversity analysis was carried out using QIIME (version 1.9.1) to evaluate the species richness and evenness within the samples (Caporaso et al., 2010). Subsequently, the Vegan 2.5.3 package in R language was used to calculate the beta distance to quantify the differences in species composition between samples (Oksanen et al., 2019). To more intuitively assess the differences between groups, the Bray-Curtis distance matrix was visualized and statistically tested using the “ggplot2” 2.2.1 package in R (Wickham, 2011). In addition, the LEfSe version 1.0 was used to mine the species biomarkers with significant differences among different groups, and to deeply analyze the biological characteristics between groups (Segata et al., 2011). Finally, the PICRUSt version 2.1.4 was applied to conduct functional prediction based on the 16S/ITS sequences (Douglas et al., 2019).

### Metabolomics analysis

The ileal samples were gradually thawed at 4°C. Then about 100 mg of the sample was added to 1 mL of pre-cooled methanol: acetonitrile (Merck,1499230-935): water solution (2:2:1, v/v). The resulting mixture was vortexed, Two low-temperature sonication treatments were performed for 30 min each (Sonics VCX130 Ultrasonic Cell Disruptor, Sonics, United States); the supernatant was subsequently left at -20°C for 60 min and then centrifuge at 13,000 × g for 15 min at 4°C in a low-temperature high-speed centrifuge (Eppendorf 5430R). The supernatant was vacuum-dried (SOLIDMIX VST Vacuum Dryer, EKATO, Germany). For MS analysis, 100 μL of an acetonitrile aqueous solution (with a ratio of acetonitrile to water of 1:1, v/v) was added to redissolve the vacuum-dried extract. This was vortexed and centrifuged at 14,000 × g and 4°C for 15 min. The supernatant was taken for sample injection analysis.

After metabolite extraction, liquid chromatography coupled with tandem MS (LC-MS/MS) was carried out. Samples were separated using an Agilent 1290 Infinity LC ultra-high-performance liquid chromatography system (Agilent Technologies) with a HILIC column (ACQUITY UPLC BEH Amide 1.7 μm, 2.1 mm × 100 mm column, Waters, United States). The column temperature was set at 25°C, the flow rate was 0.5 mL/min, and the injection volume was 2 μL. The auto-sampler was maintained at 4°C. Samples were analyzed in a random order, and QC samples were inserted to monitor the system stability.

Principal component analysis (PCA) was performed using the gmodels package in R (v2.18.1). OPLS-DA analysis using the R language ropls package (Thevenot, 2016). Differential metabolites (DMs) were screened based on the degree of change in metabolite levels, as represented by the Variable Importance in the Projection (VIP) value and *P*-value. The larger the VIP value is, the more important the metabolite is in the discrimination of differences between groups and the greater its contribution to the model. Mapping of identified metabolites to Kyoto Encyclopedia of Genes and Genomes (KEGG) pathway database.^1^

### Correlation analysis between ileal microbes and metabolites

Correlation analyses of digestive enzyme activities, immune indicators, tissue morphology and fermentation parameters with ileal microbial communities or metabolites were performed using the OmicShare tool,^2^ respectively, and Spearman correlation coefficients were used to analyze the relationship between DMs screened by metabolic pathways and microbial communities, with significance thresholds set at *P* < 0.05 and corrected by FDR (FDR < 0.1), and retained significant correlations with |r| > 0.3 for subsequent network construction. Finally, correlations and network diagrams between factors were generated using the R language Psych package and the R language Vegan package.

### Statistical analysis

Data were organized with Microsoft Excel 2021. Data related to digestive enzyme indices, immunological indices, tissue morphology, fermentation parameters, and histology were analyzed by SPSS version 22.0 (IBM Corp., Chicago, Illinois, United States) software in a general linear model with multiple comparisons using Duncan’s method. The results were presented in the form of mean and standard error. The experimental *P* < 0.05 indicates significant difference.

## Results

### Analysis of ileal digestive enzyme activity

**FIGURE 1:**
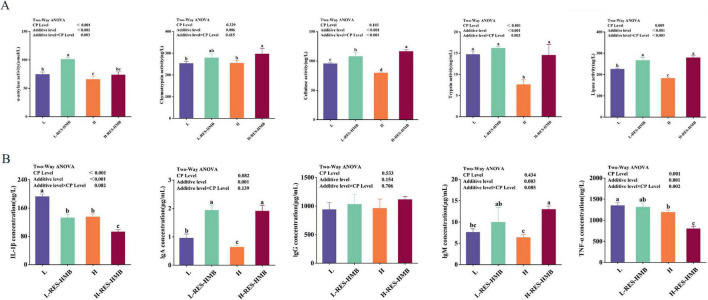
Effects of resveratrol and β-hydroxy-β-methylbutyric acid under different protein levels on the digestive enzyme activity **(A)** and immune status **(B)** of ileal contents. L: diet with 12% protein level. L-RES-HMB: diet with 12% protein level supplemented with 1.50 g/d RES and 1.25 g/d HMB. H: diet with 14% protein level. H-RES-HMB: Diet with 14% protein level supplemented with 1.50 g/d RES and 1.25 g/d HMB. IgA: Immunoglobulin A. IgM: Immunoglobulin M. IgG: Immunoglobulin G. TNF-α: tumor necrosis factor-α. IL-1β: Interleukin-1β. Different letters on the shoulder mark indicate significant difference (*P* < 0.05), the same letter or no letter indicates that the difference is not significant (*P* ≥ 0.05).

Figure 1A depicted the effects of the different experimental treatments on ileal enzymes Notably, α-amylase, trypsin, and lipase levels were significantly lower in the high-protein groups (H and H-RES-HMB) than in the corresponding low-protein groups (L and L-RES-HMB) (*P* < 0.05), indicating that the CP level affects digestive enzyme activity. Meanwhile, the groups receiving RES and HMB supplementation (L-RES-HMB and H-RES-HMB) had significantly higher α-amylase, trypsin, chymotrypsin, lipase, and cellulase activity than the corresponding non-supplemented groups (L and H, respectively) (*P* < 0.05). The CP level and RES and HMB supplementation exerted a significant interaction effect on α-amylase, trypsin, lipase, and cellulase activity (*P* < 0.05).

### Analysis of ileal immune indexes

The CP level and RES and HMB supplementation had a significant interaction effect on TNF-α levels (*P* < 0.05). The groups receiving RES and HMB supplementation (L-RES-HMB and H-RES-HMB) had significantly higher contents of IgA and IgM than the groups receiving no dietary supplementation (L and H) (*P* < 0.05). Conversely, the levels of TNF-α and IL-1β were significantly lower in the L-RES-HMB and H-RES-HMB groups than in the non-supplemented groups (*P* < 0.05). This indicates that RES and HMB supplementation has a positive effect on the immune status of the ileum in Tibetan sheep (Figure 1B).

### Analysis of mucosal morphology in the ileum

**FIGURE 2:**
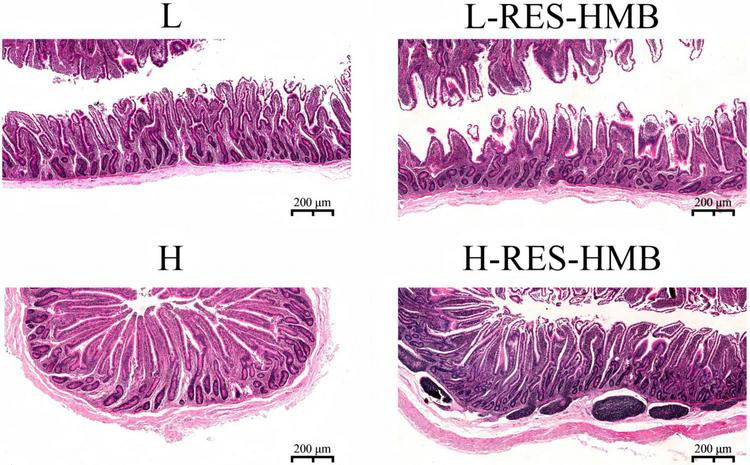
Effects of resveratrol and β-hydroxy-β-methylbutyric acid under different protein levels on the ileum morphology. Representative histological images of ileum slides stained with hematoxylin-eosin (200×).

Images showing the histopathological staining of the ileal mucosa are presented in Figure 2. As shown in Table 2, the CP level and RES and HMB supplementation exerted a significant interaction effect on the villus height, crypt depth, and mucosal thickness in the ileum (*P* < 0.05). The villus height and V/C ratio were significantly higher in the groups receiving RES and HMB supplementation than in the non-supplemented groups (*P* < 0.05).

**TABLE 2:** Effects of RES and HMB on ileal intestinal morphology in Tibetan sheep under different protein levels.

		Villus height (μm)	Villus width (μm)	Crypt depth (μm)	Mucosal thickness (μm)	Muscle layer thickness (μm)	V/C
Groups	L	440.00 ± 36.00^b^	146.00 ± 5.00	449.50 ± 61.50^a^	889.50 ± 97.50^bc^	195.50 ± 80.50	0.99 ± 0.05^bc^
	L-RES-HMB	433.00 ± 15.00^b^	150.00 ± 7.00	360.50 ± 52.50^b^	793.50 ± 67.50^c^	216.50 ± 1.50	1.23 ± 0.14^a^
	H	445.00 ± 49.51^b^	152.5 ± 6.50	485.33 ± 20.79^a^	930.33 ± 45.28^b^	243.67 ± 68.39	0.92 ± 0.13^c^
	H-RES-HMB	606.51 ± 12.50^a^	152.67 ± 7.64	517.50 ± 26.50^a^	1124.00 ± 39.00^a^	191.00 ± 5.00	1.17 ± 0.04^ab^
CP Level	LCP	436.50 ± 24.96^b^	148.00 ± 5.87	405.00 ± 70.65^b^	841.50 ± 91.60^b^	206.00 ± 52.20	1.11 ± 0.16
	HCP	525.75 ± 94.17^a^	152.58 ± 6.34	501.42 ± 27.64^a^	1027.17 ± 112.61^a^	217.33 ± 52.09	1.05 ± 0.16
Additive level	N-RES-HMB	442.50 ± 38.81^b^	149.25 ± 6.29	467.42 ± 45.51	909.92 ± 71.57	219.58 ± 71.83	0.95 ± 0.09^b^
	RES-HMB	519.75 ± 95.83^a^	151.33 ± 6.71	439.00 ± 93.69	958.75 ± 187.62	203.75 ± 14.35	1.20 ± 0.10^a^
*P*-value	CP Level	0.001	0.264	0.005	0.001	0.72	0.328
	Addictive level	0.003	0.6	0.294	0.238	0.618	0.003
	CP level x additive level	0.002	0.629	0.043	0.005	0.262	0.911

L, The dietary group with only 12% crude protein level; H, The dietary group with only 14% crude protein level; CP, Crude protein; LCP, The group with low crude protein level (12% CP); HCP, The group with high crude protein level (14% CP). L-RES-HMB, The dietary group with 12% crude protein level supplemented with 1.5 g/d of RES and 1.25 g/d of HMB; H-RES-HMB, The dietary group with 14% crude protein level supplemented with 1.5 g/d of RES and 1.25 g/d of HMB. N-RES-HMB: The dietary group without supplementation of RES and HMB. RES-HMB: The dietary group supplemented with 1.5 g/d of RES and 1.25 g/d of HMB. V/C: villus height/crypt depth ratio. Data in the same column with different letters indicate significant differences (*P* < 0.05), and data with the same letter or without a letter indicate no significant differences (*P* ≥ 0.05). The same below.

### Analysis of the SCFAs content in the ileum

There was no significant interaction effect between the CP level and RES and HMB supplementation (*P* > 0.05) (Table 3). Compared with the groups without RES and HMB supplementation (L and H), the groups receiving RES and HMB supplementation (L-RES-HMB and H-RES-HMB) showed significantly increased butyric acid levels (*P* < 0.05). Among these groups, the H-RES-HMB group showed the highest butyric acid concentration.

**TABLE 3:** Effect of RES and HMB on short chain fatty acids in the ileum of Tibetan sheep under different protein levels (%).

		Acetic acid	Propionic acid	Isobutyric acid	Butyric acid	Isovaleric acid	Valeric acid	Hexanoic acid
Groups	L	82.84 ± 0.76	5.94 ± 0.34	2.23 ± 0.52	5.45 ± 0.26^b^	2.06 ± 0.42	0.35 ± 0.27	0.13 ± 0.20
	L-RES-HMB	82.73 ± 0.44	6.17 ± 0.46	1.64 ± 0.43	6.65 ± 0.31^a^	1.76 ± 0.74	0.27 ± 0.20	0.77 ± 0.54
	H	82.77 ± 0.43	6.88 ± 0.38	1.96 ± 0.27	5.21 ± 0.36^b^	1.88 ± 0.64	0.13 ± 0.03	1.17 ± 0.20
	H-RES-HMB	82.06 ± 0.45	6.41 ± 0.61	1.43 ± 0.55	7.08 ± 0.25^a^	2.02 ± 0.26	0.17 ± 0.04	0.82 ± 0.13
CP level	LCP	82.79 ± 0.56	6.05 ± 0.39	1.94 ± 0.53	6.05 ± 0.71	1.91 ± 0.33	0.31 ± 0.22	0.95 ± 0.41
	HCP	82.42 ± 0.55	6.65 ± 0.52	1.69 ± 0.48	6.15 ± 1.06	1.95 ± 0.19	0.15 ± 0.04	0.99 ± 0.24
Additive level	N-RES-HMB	82.81 ± 0.55	6.41 ± 0.61	2.09 ± 0.40	5.33 ± 0.31^b^	1.97 ± 0.29	0.24 ± 0.21	1.15 ± 0.18
	RES-HMB	82.40 ± 0.54	6.29 ± 0.50	1.54 ± 0.46	6.87 ± 0.35^a^	1.89 ± 0.24	0.22 ± 0.14	0.80 ± 0.35
*P*-value	CP level	0.268	0.058	0.379	0.597	0.803	0.164	0.822
	Addictive level	0.220	0.679	0.067	<0.001	0.589	0.830	0.087
	CP level x Additive level	0.363	0.223	0.915	0.088	0.180	0.536	0.980

### Diversity of ileal microbiota

**FIGURE 3:**
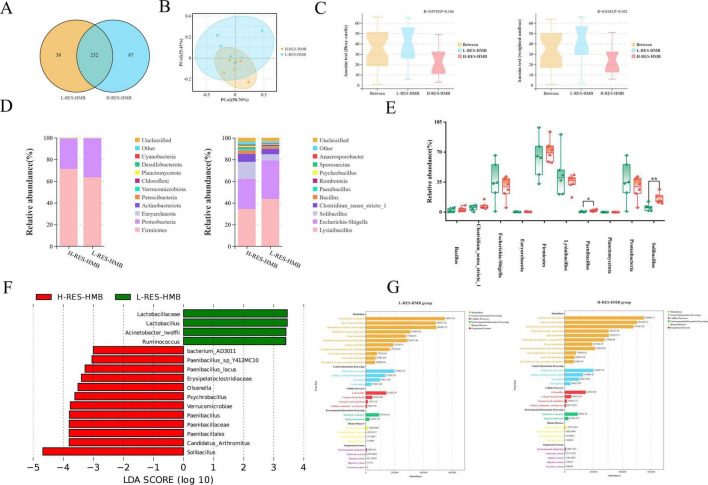
Effects of resveratrol and β-hydroxy-β-methylbutyric acid under different protein levels on the ileal microbiology. **(A)** OTU Venn diagram of the overlap of ileum microbiota. **(B)** Principal coordinate analysis (PCoA) of bacterial communities. **(C)** Anosim analysis in the ileum contents of Tibetan sheep. **(D)** Relative abundance of microbial community proportion at the phylum and genus levels. **(E)** Different bacterial phylum and genus between groups, green for L-RES-HMB group, red for H-RES-HMB group. *P < 0.05, **P < 0.01. **(F)** Linear discriminant analysis effect size (LEfSe). **(G)** KEGG function prediction.

The Venn diagram (Figure 3A) indicated that the number of operational taxonomic units (OTUs) in the H-RES-HMB group (27.27%) was higher than that in the L-RES-HMB group (14.07%), and the number of OTUs shared between the two groups was 232. Principal coordinate analysis (PCoA) revealed good clustering in the β-diversity of the ileal microbiota in the L-RES-HMB and H-RES-HMB groups (Figure 3B). Analysis of similarities (ANOSIM) based on Bray–Curtis distances yielded values of *R* = 0.0759 and *P* = 0.166, while ANOSIM based on weighted UniFrac distances yielded values of *R* = 0.0185 and *P* = 0.362 (Figure 3C). Analysis of variance showed that intergroup differences were large and greater than intragroup differences. When examining the community structure (Table 4), no significant differences in Sobs, Shannon, Simpson, Chao1, and Ace indices were observed among the ileal bacteria of the four groups.

**TABLE 4:** Alpha diversity in the ileum of Tibetan sheep between L-RES-HMB and H-RES-HMB groups.

Items	Groups		*P*-value
	L-RES-HMB	H-RES-HMB	
Sobs	307.67 ± 43.18	351.33 ± 44.63	0.116
Shannon	2.47 ± 0.77	3.13 ± 0.35	0.085
Simpson	0.62 ± 0.20	0.78 ± 0.03	0.134
Chao 1	388.03 ± 54.69	438.15 ± 51.85	0.124
Ace	408.30 ± 52.84	446.31 ± 43.72	0.204

### Microbial composition of the ileal microbiota

The relative abundances of the top 10 bacteria at the phylum and genus levels are presented in Figure 3D. At the phylum level, Firmicutes was the dominant phylum in the ileum, with the H-RES-HMB group having the highest proportion of these bacteria (71.1%). However, There was no significant difference in the relative abundance of Firmicutes between the H-RES-HMB group and the L-RES-HMB group. Similarly, there was also no obvious difference in the relative abundance of Euryarchaeota between the two groups. Additionally, the abundance of Planctomycetota in the H-RES-HMB group was significantly higher than that in the L-RES-HMB group (*P* < 0.05).

At the genus level, *Lysinibacillus* accounted for the highest proportion of bacteria in both the L-RES-HMB and H-RES-HMB groups, with a relative abundance of more than 34%. *Lysinibacillus* and *Escherichia-Shigella* emerged as the dominant genera in the two treatment groups. Compared with the L-RES-HMB group, the H-RES-HMB group contained a higher abundance of *Solibacillus*, *Clostridium_sensu_stricto_1*, *Bacillus*, and *Paenibacillus*. Specifically, the abundances of *Solibacillus* and *Paenibacillus* were significantly higher (*P* < 0.05) (Figure 3E). LEfSe analysis identified three genera enriched in two treatments (Figure 3F). The H-RES-HMB group was significantly enriched with *Solibacillus*, *Paenibacillus*, and *Psychrobacillus*.

### Differences in the functions of ileal microorganisms in Tibetan sheep

The results demonstrated that, in the four groups, the functions of microorganisms were mainly enriched in carbohydrate metabolism, amino acid metabolism, and pathways related to the metabolism of cofactors and vitamins (Figure 3G).

### Metabolic variation analysis

**FIGURE 4:**
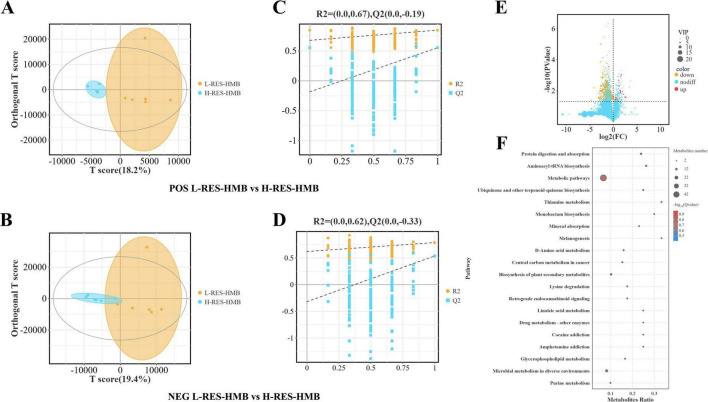
Effects of resveratrol and β-hydroxy-β-methylbutyric acid under different protein levels on the ileal metabolite. **(A)** Positive ion OPLS-DA score plots. **(B)** Negative ion OPLS-DA score plots. **(C)** Permutation test plots of positive ions. **(D)** Permutation test plots of negative ions. **(E)** Volcano plots of differential metabolites. **(F)** Analysis of KEGG pathway. The size of the bubbles indicates the number of differential metabolites enriched in the pathway, while the color of bubbles indicates the significance of enrichment in the pathway. The larger the value, the more significant the enrichment. up: significantly upregulated metabolites. nodiff: no significantly different metabolites. down: significantly downregulated metabolites.

The OPLS-DA score plot exhibited good fit (R2X and R2Y) and predictability (Q2), and there was an obvious separation between different groups. In the positive ion mode, the R2X value, R2Y value, and Q2 value of the L-RES-HMB and H-RES-HMB groups were 0.679, 0.844, and 0.550, respectively (Figure 4A). In the negative ion mode, the R2X value, R2Y value, and Q2 value of the L-RES-HMB and H-RES-HMB groups were 0.750, 0.785, and 0.533, respectively (Figure 4B). The OPLS-DA model was subjected to a displacement test. In the positive ion mode, the R2, R2X, and Q2 intercept values of the L-RES-HMB and H-RES-HMB groups were found to be 0.67, 0.00, and -0.19, respectively (Figure 4C); on the negative ion mode, these values were 0.62, 0.00, and -0.33, respectively (Figure 4D).

The analysis of DMs revealed that compared with the L-RES-HMB group, there were 229 metabolites with significant differences in abundance levels in the H-RES-HMB group (221 DMs were significantly downregulated and 18 DMs were significantly upregulated). The key up-regulated metabolites included Irinotecan, Erdosteine thioacid, 4,4′-diaminodiphenylmethane, and Morphine n-oxide (Figure 4E).

In order to identify the primary metabolic pathways and signal transduction pathways related to these DMs, KEGG pathway enrichment analysis was performed (Figure 4F). In total, the DMs between the L-RES-HMB and H-RES-HMB groups were enriched in 91 pathways. Among these pathways, 11 showed significant changes. In particular, significant differences in Protein digestion and absorption, Metabolic pathways, and Mineral absorption (*P* < 0.05) were observed. These pathways involved DMs such as L-valine, DL-tyrosine, DL-threonine, L-alanine, alpha-tocopherol, and D-xylulose. In addition, several other DMs, such as Hypoxanthine, Uric acid, and Xanthine—involved in the Purine metabolism pathway—were also identified.

### Correlation analysis

**FIGURE 5:**
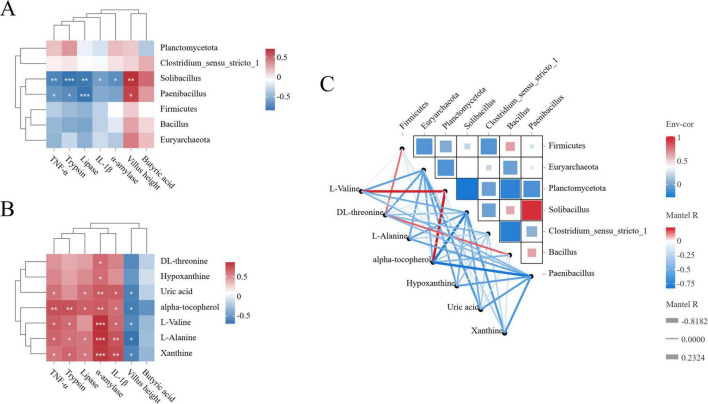
Correlation analysis. **(A)** Spearman correlation heat map of ileal bacteria and digestive enzyme activity, immune activity, ileal mucosal morphology and short-chain fatty acid concentration. **(B)** Spearman correlation heat map between ileal metabolome and digestive enzyme activity, immune activity, ileal mucosal morphology and short-chain fatty acid concentration. **(C)** Spearman correlation heat map of ileal bacteria and metabolome.

To examine the correlations among mucosal morphology in the ileum, digestive enzymes, immune activity, SCFAs, ileal microbiota, and metabolites, Spearman’s and Mantel’s correlation analyses were conducted. The Spearman correlation network revealed that the abundances of *Solibacillus* and *Paenibacillus* were positively correlated with villus height and negatively correlated with trypsin, lipase, α-amylase, and TNF-α levels in the ileum (Figure 5A). Meanwhile, the contents of L-alanine, xanthine, and alpha-tocopherol were positively correlated with lipase and trypsin activity. Additionally, xanthine, L-alanine, L-valine, alpha-tocopherol, and uric acid levels were positively correlated with IL-1β, α-amylase, and TNF-α levels and negatively correlated with villus height (Figure 5B). The abundances of Firmicutes and *Bacillus* were positively correlated with DL-threonine levels. Moreover, the abundance of Planctomycetota was positively correlated with L-valine and alpha-tocopherol levels, while that of *Solibacillus* and *Paenibacillus* was negatively correlated with alpha-tocopherol levels (Figure 5C).

## Discussion

Understanding the impact of RES and HMB on the ileum in Tibetan sheep is a valuable area of research. As a food supplement, RES can improve fish health by increasing digestive enzyme activity (Afzali-Kordmahalleh and Meshkini, 2023). It has also been demonstrated that dietary supplementation with RES can significantly elevate the activities of pepsin, α-amylase, and lipase in the duodenum and valvular intestine of Siberian sturgeon, suggesting that RES improves the digestive capacity of animals by strengthening digestive enzyme activity and subsequently increasing the feeding rate (Yang et al., 2022). Moreover, previous research has shown that adding RES and HMB to the diet can enhance the activities of trypsin, chymotrypsin, and lipase in the rumen of Tibetan sheep (Zhu et al., 2024). Consistent with these findings, the present study revealed that the L-RES-HMB and H-RES-HMB groups exhibited significantly higher activities of α-amylase, trypsin, lipase, and cellulase, thereby illustrating the positive effect of RES and HMB supplementation on digestive enzyme activity in the ileum of Tibetan sheep.

Immunoglobulins are large glycoproteins that mediate adaptive immune responses by recognizing pathogens, preventing pathogen invasion, promoting pathogen neutralization, and achieving pathogen clearance and destruction. Therefore, immunoglobulins play an important role in immune regulation and pathogen defense in animals (Keyt et al., 2020). The mucosal B cells differentiate into plasma cells to produce IgA and IgM, which resist inhaled (Frede et al., 2022), ingested, and sexually transmitted pathogens and antigens that come into contact with the mucosal surface (Kaetzel, 2005). Mechanistically, RES indirectly promotes IgA secretion by inhibiting intestinal inflammation and improving mucosal barrier microenvironment, while HMB enhances B cell class switch recombination from IgM to IgA by stimulating IL-6 and BAFF secretion from bone marrow stromal cells—*In vitro* studies show HMB increases B cell IgA secretion by 22% (Gan et al., 2024; Kornasio et al., 2009). Notably, RES also elevates serum IgG levels in weaned piglets (Chen et al., 2021), aligning with the present study’s findings.

Pro-inflammatory cytokines TNF-α and IL-1β are central regulators of inflammation (Esposito and Cuzzocrea, 2009), and RES—a plant-derived polyphenol—exhibits anti-inflammatory activity through defined molecular pathways (Novakovic et al., 2022). RES activates silent information regulator 1 (SIRT1), promoting deacetylation of NF-κB subunit p65 and inhibiting its nuclear translocation to reduce TNF-α/IL-1β transcription. For example, in TNF-α-stimulated fibroblasts, RES reduces p65 acetylation, NF-κB reporter activity, and pro-inflammatory mRNA (Malaguarnera, 2019; Zhu et al., 2011). HMB complements this by activating AMPK to inhibit IκB kinase phosphorylation, blocking IκBα degradation and suppressing NF-κB pathway activation. In ovine myoblast injury models, HMB decreases p-IKKα/β, p-IκBα, and nuclear NF-κB p65, reducing IL-6/TNF-α secretion (Arazi et al., 2018; Zheng et al., 2022). Additionally, RES reduces M1 macrophage polarization via NF-κB inhibition and PI3K/Akt activation, shown by decreased iNOS/TNF-α in obese mouse muscle and IL-1β in rat corneal transplants (Shabani et al., 2020; Xu et al., 2023), while HMB curbs TNF-α indirectly by inhibiting dendritic cell maturation and Th1 cytokine secretion (Arazi et al., 2018). Their antioxidant activities—RES scavenging free radicals and inhibiting NADPH oxidase, HMB maintaining glutathione homeostasis—synergistically suppress NF-κB-dependent oxidative stress cascades (Gan et al., 2024; Arazi et al., 2018). Consistent with prior studies (Bereswill et al., 2010; Ozcicek et al., 2016; Gan et al., 2024), this study found RES and HMB supplementation reduced ileal TNF-α/IL-1β levels independent of dietary protein. RES has shown therapeutic effects against weaning stress, heat stress, and bacterial infections (Franco et al., 2013; Cheng et al., 2019a; Cheng et al., 2019b; Gan et al., 2019), while enhancing animal growth and product quality (Meng et al., 2023). Our results highlight specific impacts on ileal immune markers (IgA, IgM, TNF-α, IL-1β), with the RES-HMB-high-protein diet demonstrating optimal effects in maintaining intestinal health and immune regulation—a coherence supported by molecular, cellular, and animal model evidence (Malaguarnera, 2019; Arazi et al., 2018).

The ileal mucosa is crucial for digestion and absorption. A good mucosal morphology can increase the absorption area of the ileum and improve the rate of nutrient utilization (Jia et al., 2022). The present study found that dietary RES and HMB supplementation could be a novel strategy for improving the mucosal morphology in the ileum of Tibetan sheep. RES shows various biological activities, such as antioxidant and anti-inflammatory effects, and may improve the mucosal morphology of the ileum by reducing intestinal inflammation and protecting mucosal cells (Nunes et al., 2018). Adding 400 mg/kg RES to the diet was found to increase the jejunal villus height and the ratio of villus height to crypt depth in heat-stressed broilers (Chen et al., 2021). It was found that RES may prevent barrier defects and inflammation by inhibiting neutrophil infiltration in mice with colitis, and RES has also been proven to inhibit TNF-α-induced inflammatory signaling and IL-8 production in Caco-2 human intestinal cells (Mayangsari and Suzuki, 2018a). Meanwhile, as a nutritional supplement in humans, HMB also exerts a positive effect on the intestinal mucosa while promoting muscle growth (Slater and Jenkins, 2000). In pigs treated with lipopolysaccharide, HMB supplementation can significantly increase the jejunal villus height and the ratio of ileal villus height to crypt depth, while reducing ileal crypt depth. In addition, HMB further ameliorates intestinal function in weaned piglets via augmenting the activities of intestinal mucosal disaccharidase and crucial tricarboxylic acid cycle enzymes (Zheng et al., 2020). The present study found that adding RES and HMB to the diet of Tibetan sheep significantly increases the V/C ratio. In addition, the villus height and mucosal thickness were found to be significantly higher in the H-RES-HMB group than in the L-RES-HMB group, indicating that the ileum had stronger digestion and absorption capabilities in the H-RES-HMB group. In line with these findings, RES-HMB supplement increased jejunal permeability and the V/C of the jejunum in sheep (Ji et al., 2024b). However, the specific effect of RES-HMB on mucosal morphology in the ileum still warrants further exploration.

SCFAs are the primary end products derived from the fermentation of indigestible carbohydrates by the intestinal microbiota (Ríos-Covián et al., 2016). The significant increase in butyric acid levels after RES and HMB supplementation was a key finding of the present study. Butyric acid plays a crucial role in maintaining the integrity of intestinal epithelial cells (Jung et al., 2015; Morrison and Preston, 2016), regulating intestinal immune responses (Peng et al., 2009), promoting colon peristalsis, and reducing inflammation (Zhang et al., 2010). Butyrate inhibits TNF-α, IL-6, and myeloperoxidase by preventing nuclear factor kappa β activation in Kupffer cells (Huang et al., 2010). Among the SCFAs produced in the colon, butyrate appears to be the most important regulator of tight junction proteins. Furthermore, it has been demonstrated to strengthen intestinal barrier function by elevating the expression of claudin-1 and zonula occludens-1 and the redistribution of occluding (Manco et al., 2010). Interestingly, RES and butyric acid can be converted into resveratrol butyrate via n-ethyl-N′-(3-dimethylaminopropyl)carbodiimide and 4-dimethylaminopyridine, and the esterification of RES increases its biological activity (Shih et al., 2021). The evidence clearly demonstrates that as a functional food ingredient, additive, or health-promoting supplement, RES may act as an anti-fat accumulation agent (Tain et al., 2020). In conclusion, RES and HMB supplementation has a complex impact on intestinal microbes, and further research is required to elucidate the specific mechanism through which RES-HMB affects the intestinal microbiota. Collectively, the above phenotypic results demonstrated that adding RES and HMB to the diet has a positive effect on the ileal health of Tibetan sheep. Subsequent omics analysis was performed to explore the ileal characteristics of Tibetan sheep in the L-RES-HMB and H-RES-HMB groups in detail.

In the microbial community of the animal ileum, the phylum of Firmicutes is an important structural component (He et al., 2018). It not only plays a key role in the construction of the community but also participates in the degradation of intestinal fibers, thus playing an important function in maintaining the balance of the intestinal microecology and nutritional metabolism (Marques et al., 2017). Most of the microorganisms that produce butyrate and are related to the human gut belong to the phylum Firmicutes (Brame et al., 2021). The bacteria in this phylum decompose dietary fiber and produce SCFAs such as acetic acid, propionic acid, and butyric acid. These SCFAs not only provide energy for intestinal cells, but also have important functions such as regulating the immune system and maintaining intestinal barrier function (Huang et al., 2018). In patients with inflammatory bowel disease (IBD), the abundance of *Faecalibacterium prausnitzii*, a bacterium belonging to the phylum Firmicutes, is very low. A reduction in the proportion of these bacteria is related to impaired intestinal mucosal protection (Bangsgaard Bendtsen et al., 2012). *Faecalibacterium prausnitzii* is a relatively abundant species within the family Ruminococcaceae and can synthesize butyrate via butyryl-CoA and acetate-CoA transferases. In Firmicutes, the acetyl-CoA pathway serves as the main route for butyrate synthesis (Louis and Flint, 2009).

The abundances of *Solibacillus* and *Paenibacillus* were found to be positively correlated with villus height in the present study. This indicated that these microbial communities may play an active role in maintaining specific structures or functions of the ileum. The number of intestinal villi determines the intestinal surface area, and these structures are connected to the circulatory system, improving the intestinal absorption capacity (Gehart and Clevers, 2019). In the study by Yu et al. (2019), which examined the effects of different CP levels, the villus height of the 20% protein diet group was found to be higher than that of the 14 and 17% protein diet groups. In addition, villus height is related to factors such as mucosal barrier function (Yamauchi, 2002). Interestingly, the relationship of *Solibacillus* and *Paenibacillus* with villus height has not been reported in previous studies and needs further investigation. However, Research confirmed that non-specific immunity is enhanced in fish receiving *Paenibacillus polymyxa* supplementation (Gupta et al., 2016). Moreover, there was reports depict the favorable impacts of bacilli like Bacillus coagulans, Bacillus licheniformis, and Paenibacillus polymyxa on the growth, immunity, and disease resistance of aquatic animals (Amoah et al., 2021). We believe that the significant increase in the abundance of *Solibacillus* and *Paenibacillus* in the ileum of Tibetan sheep may be related to the reduction in TNF-α levels, suggesting that these bacterial genera may exert a regulatory effect on specific components of the immune system.

Xanthine is a product of purine metabolism, and its presence can reflect the activity of purine metabolism in microbes. In this study, the content of Xanthine in the high-protein group was lower than that in the low-protein group, likely due to the increase in protein levels. Uric acid is an end product of purine metabolism (Granell et al., 2004), and its levels can increase due to changes in purine metabolism (Wu et al., 2020). Xanthine oxidoreductase (XOR) mainly catalyzes the conversion of hypoxanthine to xanthine and then uric acid (Battelli et al., 2018). Some studies show that RES supplementation can reduce serum uric acid levels in patients with dyslipidemia, with lower xanthine oxidase (XO, a key enzyme involved in uric acid production) activity under high RES doses (600 mg/d). Thus, there exists a dose–response relationship between the reduction in uric acid and XO activity. RES can attenuate renal inflammation and reduce blood uric acid levels in mouse models of high-fat diet-induced insulin resistance. One study using a rabbit intestinal loop infection model found that during *in vivo* enteropathogenic *Escherichia coli* infection, uric acid crystals are formed in the intestinal lumen (Crane and Mongiardo, 2014). These uric acid crystals have a pro-inflammatory effect independent of hydrogen peroxide production and can trigger IL-1β-mediated inflammation by activating the NOD-like receptor protein 3 (NLRP3) inflammasome (Yin et al., 2019). Elevated serum uric acid levels in diabetic rats are associated with the increased activity of α-amylase (Sanni et al., 2019). Xanthine and uric acid accumulation may result from the dysfunction of purine metabolism, which in turn affects the physiological function of the intestine. Additionally, studies have demonstrated that the number of butyrate-producing bacteria and the production of SCFAs are both positively correlated with the ability to reduce uric acid levels and inhibit inflammation in animals (Li et al., 2023). However, the specific mechanism still requires further investigation. In conclusion, both dietary protein levels and the addition of RES and HMB not only alter the microbial community structure of the ileum but also impact different metabolic pathways and metabolites. Nevertheless, the relationship between the microbiota and metabolites needs to be studied in further detail.

Interestingly, in the present study, Firmicutes and *Bacillus* showed high abundances in the high-protein group and were positively correlated with DL-threonine levels. The abundance of *Planctomycetota* was positively correlated with L-valine and alpha-tocopherol levels. As an essential amino acid, L-valine plays a crucial role in protein synthesis and metabolism (Zhang J. et al., 2019). Meanwhile, alpha-tocopherol has powerful physiological functions, such as antioxidation (Ley et al., 2005). The positive correlation between *Planctomycetota* and these metabolites implies that this group of bacteria is involved in their metabolism or regulation. Interestingly, we found that *Solibacillus* and *Paenibacillus* were negatively correlated with alpha-tocopherol. This may be because the metabolic activities of these microbes compete with the metabolism or utilization of alpha-tocopherol. Additionally, the antioxidant effect of alpha-tocopherol may also inhibit the growth of *Solibacillus* and *Paenibacillus* the metabolites produced by microorganisms may also affect nutrient metabolism (Nicholson et al., 2012).

In conclusion, the protein level of the feed is also important for intestinal immune regulation (Pearce et al., 2024). Different intestinal microbes and metabolites may activate or inhibit the intestinal immune system in the host. In this study, compared with the L-RES-HMB treatment, the H-RES-HMB treatment exerted a stronger complementary effect on the ileum of Tibetan sheep. In the future, we plan to further explore the effects of different protein sources and formulations on the intestinal microbiota and metabolites in sheep in order to improve the intestinal health and production performance of animals by adjusting feed protein levels.

## Conclusion

The results of this study indicate that a 14% protein diet can significantly increase the concentration of butyric acid in the ileum of Tibetan sheep, thus improving ileal morphological development and function. The addition of RES and HMB leads to a further improvement in the health of the ileum. This improvement can be attributed to the combined action of protein level and RES and HMB supplementation, which positively influence the ileal microbial community composition (Firmicutes and *Clostridium_sensu_stricto_1*) and metabolite levels (xanthine and uric acid), thereby enhancing butyric acid levels. This increase leads to improved digestive enzyme activity, immune responses, and mucosal morphology, ultimately promoting the health of the ileum in Tibetan sheep.

## Data Availability

The original contributions presented in the study are publicly available. This data can be found here: NCBI SRA (accession: PRJNA1276720) and OMIX (accession: PRJCA041554).

## Footnotes

1
http://www.genome.ad.jp/kegg/

2
https://www.omicshare.com

## References

[B1] Afzali-Kordmahalleh A. Meshkini S. (2023). Effects of dietary resveratrol supplementation on digestive enzymes activities and serum biochemistry of rainbow trout (*Oncorhynchus mykiss*). *Vet. Res. Forum.* 14, 625–630. 10.30466/vrf.2023.560224.3650 38169480 PMC10758009

[B2] Amoah K. Dong X.-H. Tan B.-P. Zhang S. Chi S.-Y. Yang Q.-H. (2021). Effects of three probiotic strains (Bacillus coagulans, B. licheniformis and Paenibacillus polymyxa) on growth, immune response, gut morphology and microbiota, and resistance against Vibrio harveyi of northern whitings, Sillago sihama Forsskál (1775). *Anim. Feed Sci. Technol.* 277:114958. 10.1016/j.anifeedsci.2021.114958

[B3] Arazi H. Taati B. Suzuki K. (2018). A review of the effects of leucine metabolite (β-hydroxy-β-methylbutyrate) supplementation and resistance training on inflammatory markers: A new approach to oxidative stress and cardiovascular risk factors. *Antioxidants* 7:148. 10.3390/antiox7100148 30347824 PMC6210682

[B4] Bangsgaard Bendtsen K. M. Krych L. Sørensen D. B. Pang W. Nielsen D. S. Josefsen K. (2012). Gut microbiota composition is correlated to grid floor induced stress and behavior in the BALB/c mouse. *PLoS One* 7:e46231. 10.1371/journal.pone.0046231 23056268 PMC3462757

[B5] Battelli M. G. Bortolotti M. Polito L. Bolognesi A. (2018). The role of xanthine oxidoreductase and uric acid in metabolic syndrome. *Biochim. Biophys. Acta (BBA) Mol. Basis Dis.* 1864 2557–2565. 10.1016/j.bbadis.2018.05.003 29733945

[B6] Bereswill S. Muñoz M. Fischer A. Plickert R. Haag L.-M. Otto B. (2010). Anti-inflammatory effects of resveratrol, curcumin and simvastatin in acute small intestinal inflammation. *PLoS One* 5:e15099. 10.1371/journal.pone.0015099 21151942 PMC2997083

[B7] Bianchi F. Dall’asta M. Del Rio D. Mangia A. Musci M. Scazzina F. (2011). Development of a headspace solid-phase microextraction gas chromatography–mass spectrometric method for the determination of short-chain fatty acids from intestinal fermentation. *Food Chem.* 129 200–205. 10.1016/j.foodchem.2011.04.022

[B8] Bokulich N. A. Subramanian S. Faith J. J. Gevers D. Gordon J. I. Knight R. (2013). Quality-filtering vastly improves diversity estimates from Illumina amplicon sequencing. *Nat. Methods* 10 57–59. 10.1038/nmeth.2276 23202435 PMC3531572

[B9] Brame J. E. Liddicoat C. Abbott C. A. Breed M. F. (2021). The potential of outdoor environments to supply beneficial butyrate-producing bacteria to humans. *Sci. Total Environ.* 777:146063. 10.1016/j.scitotenv.2021.146063 33684759

[B10] Caporaso J. G. Kuczynski J. Stombaugh J. Bittinger K. Bushman F. D. Costello E. K. (2010). QIIME allows analysis of high-throughput community sequencing data. *Nat. Methods* 7 335–336. 10.1038/nmeth.f.303 20383131 PMC3156573

[B11] Chen D. Chen X. Tu Y. Wang B. Lou C. Ma T. (2015). Effects of mulberry leaf flavonoid and resveratrol on methane emission and nutrient digestion in sheep. *Anim. Nutr.* 1 362–367. 10.1016/j.aninu.2015.12.008 29767046 PMC5940990

[B12] Chen S. Zhou Y. Chen Y. Gu J. (2018). fastp: An ultra-fast all-in-one FASTQ preprocessor. *Bioinformatics* 34 i884–i890. 10.1093/bioinformatics/bty560 30423086 PMC6129281

[B13] Chen X. Zeng Z. Huang Z. Chen D. He J. Chen H. (2021). Effects of dietary resveratrol supplementation on immunity, antioxidative capacity and intestinal barrier function in weaning piglets. *Anim. Biotechnol.* 32 240–245. 10.1080/10495398.2019.1683022 31645181

[B14] Cheng K. Song Z. Li S. Yan E. Zhang H. Zhang L. (2019a). Effects of resveratrol on intestinal oxidative status and inflammation in heat-stressed rats. *J. Thermal Biol.* 85:102415. 10.1016/j.jtherbio.2019.102415 31657756

[B15] Cheng K. Yan E. Song Z. Li S. Zhang H. Zhang L. (2019b). Protective effect of resveratrol against hepatic damage induced by heat stress in a rat model is associated with the regulation of oxidative stress and inflammation. *J. Thermal Biol.* 82 70–75. 10.1016/j.jtherbio.2019.03.012 31128661

[B16] Crane J. K. Mongiardo K. M. (2014). Pro-inflammatory effects of uric acid in the gastrointestinal tract. *Immunol. Invest.* 43 255–266. 10.3109/08820139.2013.864667 24377830 PMC3954906

[B17] Douglas G. M. Maffei V. J. Zaneveld J. Yurgel S. N. Brown J. R. Taylor C. M. (2019). PICRUSt2: An improved and extensible approach for metagenome inference. *BioRxiv [PrePrint]* 10.1101/672295

[B18] Duan Y. Zhong Y. Xiao H. Zheng C. Song B. Wang W. (2019). Gut microbiota mediates the protective effects of dietary β−hydroxy−β−methylbutyrate (HMB) against obesity induced by high-fat diets. *FASEB J.* 33 10019–10033. 10.1096/fj.201900665RR 31167080

[B19] Edgar R. C. (2010). Search and clustering orders of magnitude faster than BLAST. *Bioinformatics* 26 2460–2461. 10.1093/bioinformatics/btq461 20709691

[B20] Edgar R. C. Haas B. J. Clemente J. C. Quince C. Knight R. (2011). UCHIME improves sensitivity and speed of chimera detection. *Bioinformatics* 27 2194–2200. 10.1093/bioinformatics/btr381 21700674 PMC3150044

[B21] Esposito E. Cuzzocrea S. (2009). TNF-alpha as a therapeutic target in inflammatory diseases, ischemia-reperfusion injury and trauma. *Curr. Med. Chem.* 16 3152–3167. 10.2174/092986709788803024 19689289

[B22] Franco J. G. Lisboa P. C. Lima N. S. Amaral T. A. Peixoto-Silva N. Resende A. C. (2013). Resveratrol attenuates oxidative stress and prevents steatosis and hypertension in obese rats programmed by early weaning. *J. Nutr. Biochem.* 24 960–966. 10.1016/j.jnutbio.2012.06.019 22959054

[B23] Frede A. Czarnewski P. Monasterio G. Tripathi K. P. Bejarano D. A. Flores R. O. R. (2022). B cell expansion hinders the stroma-epithelium regenerative cross talk during mucosal healing. *Immunity* 55 2336–2351.e12. 10.1016/j.immuni.2022.11.002 36462502

[B24] Gan J. Ji Q. Su Q. Hou S. Gui L. (2024). Resveratrol and β-hydroxy-β-methylbutyric acid supplementation promotes ileal development and digestive function by altering microbial community abundance and metabolites in Tibetan sheep. *Front. Vet. Sci.* 11:1470992. 10.3389/fvets.2024.1470992 39723186 PMC11668758

[B25] Gan Z. Wei W. Li Y. Wu J. Zhao Y. Zhang L. (2019). Curcumin and resveratrol regulate intestinal bacteria and alleviate intestinal inflammation in weaned piglets. *Molecules* 24:1220. 10.3390/molecules24071220 30925757 PMC6479679

[B26] Gehart H. Clevers H. (2019). Tales from the crypt: New insights into intestinal stem cells. *Nat. Rev. Gastroenterol. Hepatol.* 16 19–34. 10.1038/s41575-018-0081-y 30429586

[B27] Granell S. Bulbena O. Genesca M. Sabater L. Sastre J. Gelpi E. (2004). Mobilization of xanthine oxidase from the gastrointestinal tract in acute pancreatitis. *BMC Gastroenterol.* 4:1. 10.1186/1471-230X-4-1 14728722 PMC331409

[B28] Gupta A. Gupta P. Dhawan A. (2016). Paenibacillus polymyxa as a water additive improved immune response of Cyprinus carpio and disease resistance against Aeromonas hydrophila. *Aquacult. Rep.* 4 86–92. 10.1016/j.aqrep.2016.07.002

[B29] He J. Yi L. Hai L. Ming L. Gao W. Ji R. (2018). Characterizing the bacterial microbiota in different gastrointestinal tract segments of the Bactrian camel. *Sci. Rep.* 8:654. 10.1038/s41598-017-18298-7 29330494 PMC5766590

[B30] Huang W. Metlakunta A. Dedousis N. Zhang P. Sipula I. Dube J. J. (2010). Depletion of liver Kupffer cells prevents the development of diet-induced hepatic steatosis and insulin resistance. *Diabetes* 59 347–357. 10.2337/db09-0016 19934001 PMC2809951

[B31] Huang Y. Shi X. Li Z. Shen Y. Shi X. Wang L. (2018). Possible association of Firmicutes in the gut microbiota of patients with major depressive disorder. *Neuropsychiatric Dis. Treat.* 14 3329–3337. 10.2147/NDT.S188340 30584306 PMC6284853

[B32] Ji Q. Zhang F. Su Q. He T. Wu Z. Zhu K. (2024a). Effect of supplementing lysins and methionine to low-protein diets on growth performance, hepatic antioxidant capacity, immune status, and glycolytic activity of tibetan sheep. *BMC Genomics* 25:557. 10.1186/s12864-024-10480-2 38834972 PMC11149200

[B33] Ji Q. Zhang F. Zhang Y. Su Q. He T. Hou S. (2024b). Multi-omics revealed resveratrol and β-Hydroxy-β-methyl butyric acid alone or in combination improved the jejunal function in tibetan sheep. *Antioxidants* 13:892. 10.3390/antiox13080892 39199138 PMC11351831

[B34] Jia L. Wu J. Lei Y. Kong F. Zhang R. Sun J. (2022). Oregano essential oils mediated intestinal microbiota and metabolites and improved growth performance and intestinal barrier function in sheep. *Front. Immunol.* 13:908015. 10.3389/fimmu.2022.908015 35903106 PMC9314563

[B35] Jung T.-H. Park J. H. Jeon W.-M. Han K.-S. (2015). Butyrate modulates bacterial adherence on LS174T human colorectal cells by stimulating mucin secretion and MAPK signaling pathway. *Nutr. Res. Pract.* 9 343–349. 10.4162/nrp.2015.9.4.343 26244071 PMC4523476

[B36] Kaetzel C. S. (2005). The polymeric immunoglobulin receptor: Bridging innate and adaptive immune responses at mucosal surfaces. *Immunol. Rev.* 206 83–99. 10.1111/j.0105-2896.2005.00278.x 16048543

[B37] Keyt B. Baliga R. Sinclair A. Carroll S. Peterson M. (2020). Structure, function, and therapeutic use of IgM antibodies. *Antibodies* 9:53. 10.3390/antib9040053 33066119 PMC7709107

[B38] Kop Bozbay C. Yilmaz B. Ocak N. (2024). Beta-hydroxy−β−methyl butyrate-supplemented diet for broiler chickens is more conducive to dietary protein reduction than a leucine-supplemented diet until 21 days old. *J. Sci. Food Agric.* 104 1450–1457. 10.1002/jsfa.13023 37800278

[B39] Kornasio R. Riederer I. Butler-Browne G. Mouly V. Uni Z. Halevy O. (2009). β-hydroxy-β-methylbutyrate (HMB) stimulates myogenic cell proliferation, differentiation and survival via the MAPK/ERK and PI3K/Akt pathways. *Biochim. Biophys. Acta* 1793 755–763. 10.1016/j.bbamcr.2008.12.017 19211028

[B40] Ley R. E. Bäckhed F. Turnbaugh P. Lozupone C. A. Knight R. D. Gordon J. I. (2005). Obesity alters gut microbial ecology. *Proc. Natl. Acad. Sci. U S A.* 102 11070–11075. 10.1073/pnas.0504978102 16033867 PMC1176910

[B41] Li Y. Li H. Wang R. Yu Y. Liu X. Tian Z. (2023). Protective effect of sodium butyrate on intestinal barrier damage and uric acid reduction in hyperuricemia mice. *Biomed. Pharmacother.* 161:114568. 10.1016/j.biopha.2023.114568 36948133

[B42] Lotti C. Rubert J. Fava F. Tuohy K. Mattivi F. Vrhovsek U. (2017). Development of a fast and cost-effective gas chromatography–mass spectrometry method for the quantification of short-chain and medium-chain fatty acids in human biofluids. *Anal. Bioanal. Chem.* 409 5555–5567. 10.1007/s00216-017-0493-5 28717897

[B43] Louis P. Flint H. J. (2009). Diversity, metabolism and microbial ecology of butyrate-producing bacteria from the human large intestine. *FEMS Microbiol. Lett.* 294 1–8. 10.1111/j.1574-6968.2009.01514.x 19222573

[B44] Ma T. Chen D. D. Tu Y. Zhang N. F. Si B. W. Deng K. D. (2015). Effect of dietary supplementation with resveratrol on nutrient digestibility, methanogenesis and ruminal microbial flora in sheep. *J. Anim. Physiol. Anim. Nutr.* 99 676–683. 10.1111/jpn.12264 25319536

[B45] Ma Y. Han L. Raza S. H. A. Gui L. Zhang X. Hou S. (2023). Exploring the effects of palm kernel meal feeding on the meat quality and rumen microorganisms of Qinghai Tibetan sheep. *Food Sci. Nutr.* 11 3516–3534. 10.1002/fsn3.3340 37324863 PMC10261763

[B46] Magoc T. Salzberg S. L. (2011). FLASH: Fast length adjustment of short reads to improve genome assemblies. *Bioinformatics* 27 2957–2963. 10.1093/bioinformatics/btr507 21903629 PMC3198573

[B47] Malaguarnera L. (2019). Influence of resveratrol on the immune response. *Nutrients* 11:946. 10.3390/nu11050946 31035454 PMC6566902

[B48] Manco M. Putignani L. Bottazzo G. F. (2010). Gut microbiota, lipopolysaccharides, and innate immunity in the pathogenesis of obesity and cardiovascular risk. *Endocrine Rev.* 31 817–844. 10.1210/er.2009-0030 20592272

[B49] Marques F. Z. Nelson E. Chu P.-Y. Horlock D. Fiedler A. Ziemann M. (2017). High-fiber diet and acetate supplementation change the gut microbiota and prevent the development of hypertension and heart failure in hypertensive mice. *Circulation* 135 964–977. 10.1161/CIRCULATIONAHA.116.024545 27927713

[B50] Mayangsari Y. Suzuki T. (2018a). Resveratrol ameliorates intestinal barrier defects and inflammation in colitic mice and intestinal cells. *Journal of Agricultural and Food Chemistry* 66 12666–12674. 10.1021/acs.jafc.8b04138 30426751

[B51] Mayangsari Y. Suzuki T. (2018b). Resveratrol enhances intestinal barrier function by ameliorating barrier disruption in Caco-2 cell monolayers. *J. Funct. Foods* 51 39–46. 10.1016/j.jff.2018.10.009

[B52] Meng Q. Li J. Wang C. Shan A. (2023). Biological function of resveratrol and its application in animal production: A review. *J. Anim. Sci. Biotechnol.* 14:25. 10.1186/s40104-022-00822-z 36765425 PMC9921422

[B53] Morrison D. J. Preston T. (2016). Formation of short chain fatty acids by the gut microbiota and their impact on human metabolism. *Gut Microbes* 7 189–200. 10.1080/19490976.2015.1134082 26963409 PMC4939913

[B54] Nicholson J. K. Holmes E. Kinross J. Burcelin R. Gibson G. Jia W. (2012). Host-gut microbiota metabolic interactions. *Science* 336 1262–1267. 10.1126/science.1223813 22674330

[B55] Novakovic R. Rajkovic J. Gostimirovic M. Gojkovic-Bukarica L. Radunovic N. (2022). resveratrol and reproductive health. *Life* 12:294. 10.3390/life12020294 35207581 PMC8875092

[B56] Nunes S. Danesi F. del Rio D. Silva P. (2018). Resveratrol and inflammatory bowel disease: The evidence so far. *Nutr. Res. Rev.* 31 85–97. 10.1017/S095442241700021X 29191255

[B57] Oksanen J. Blanchet F. G. Friendly M. Kindt R. Legendre P. Mcglinn D. (2019). *vegan: Community Ecology Package. R Package Version 2.5-6.*

[B58] Ozcicek A. Cetin N. Keskin Cimen F. Tumkaya L. Malkoc I. Gulaboglu M. (2016). The impact of resveratrol on oxidative stress induced by methotrexate in rat ileum tissue: Evaluation of biochemical and histopathological features and analysis of gene expression. *Med. Princ. Pract.* 25 181–186. 10.1159/000442020 26517535 PMC5588348

[B59] Pasquariello R. Verdile N. Brevini T. A. Gandolfi F. Boiti C. Zerani M. (2020). The role of resveratrol in mammalian reproduction. *Molecules* 25:4554. 10.3390/molecules25194554 33027994 PMC7582294

[B60] Pearce S. C. Nisley M. J. Kerr B. J. Sparks C. Gabler N. K. (2024). Effects of dietary protein level on intestinal function and inflammation in nursery pigs. *J. Anim. Sci.* 102:skae077. 10.1093/jas/skae077 38504643 PMC11015048

[B61] Peng L. Li Z.-R. Green R. S. Holzmanr I. R. Lin J. (2009). Butyrate enhances the intestinal barrier by facilitating tight junction assembly via activation of AMP-activated protein kinase in Caco-2 cell monolayers. *J. Nutr.* 139 1619–1625. 10.3945/jn.109.104638 19625695 PMC2728689

[B62] Ríos-Covián D. Ruas-Madiedo P. Margolles A. Gueimonde M. De Los Reyes-Gavilán C. G. Salazar N. (2016). Intestinal short chain fatty acids and their link with diet and human health. *Front. Microbiol.* 7:185. 10.3389/fmicb.2016.00185 26925050 PMC4756104

[B63] Sanni O. Erukainure O. L. Oyebode O. Islam M. S. (2019). Anti-hyperglycemic and ameliorative effect of concentrated hot water-infusion of Phragmanthera incana leaves on type 2 diabetes and indices of complications in diabetic rats. *J. Diabetes Metab. Disord.* 18 495–503. 10.1007/s40200-019-00456-5 31890675 PMC6914750

[B64] Segata N. Izard J. Waldron L. Gevers D. Miropolsky L. Garrett W. S. (2011). Metagenomic biomarker discovery and explanation. *Genome Biol.* 12:R60. 10.1186/gb-2011-12-6-r60 21702898 PMC3218848

[B65] Sha Y. Ren Y. Zhao S. He Y. Guo X. Pu X. (2022). Response of ruminal microbiota–host gene interaction to high-altitude environments in Tibetan sheep. *Int. J. Mol. Sci.* 23:12430. 10.3390/ijms232012430 36293284 PMC9604387

[B66] Shabani M. Sadeghi A. Hosseini H. Teimouri M. Babaei Khorzoughi R. Pasalar P. (2020). Resveratrol alleviates obesity-induced skeletal muscle inflammation via decreasing M1 macrophage polarization and increasing the regulatory T cell population. *Sci. Rep.* 10:3791. 10.1038/s41598-020-60185-1 32123188 PMC7052230

[B67] Shih M.-K. Tain Y.-L. Cheng C.-M. Hsu C.-N. Chen Y.-W. Huang H.-T. (2021). Separation and identification of resveratrol butyrate ester complexes and their bioactivity in HepG2 cell models. *Int. J. Mol. Sci.* 22:13539. 10.3390/ijms222413539 34948341 PMC8703675

[B68] Slater G. J. Jenkins D. (2000). β-hydroxy-β-methylbutyrate (HMB) supplementation and the promotion of muscle growth and strength. *Sports Med.* 30 105–116. 10.2165/00007256-200030020-00004 10966150

[B69] Tain Y.-L. Jheng L.-C. Chang S. K. Chen Y.-W. Huang L.-T. Liao J.-X. (2020). Synthesis and characterization of novel resveratrol butyrate esters that have the ability to prevent fat accumulation in a liver cell culture model. *Molecules* 25:4199. 10.3390/molecules25184199 32937766 PMC7571132

[B70] Thevenot E. (2016). *PCA, PLS (-DA) and OPLS (-DA) for Multivariate Analysis and Feature Selection of Omics Data.* 1, 354–405. 10.1021/acs.jproteome.5b00354 26088811

[B71] Wang C. Zhao F. Li Z. Jin X. Chen X. Geng Z. (2021). Effects of resveratrol on growth performance, intestinal development, and antioxidant status of broilers under heat stress. *Animals* 11:1427. 10.3390/ani11051427 34067505 PMC8155960

[B72] Wang Q. Zeng Y. Zeng X. Wang X. Wang Y. Dai C. (2021). Effects of dietary energy levels on rumen fermentation, gastrointestinal tract histology, and bacterial community diversity in fattening male Hu lambs. *Front. Microbiol.* 12:695445. 10.3389/fmicb.2021.695445 34566905 PMC8460862

[B73] Wickham H. (2011). ggplot2. *Wiley Interdiscipl. Rev. Comput. Stat.* 3 180–185. 10.1002/wics.147

[B74] Wu J. Wei Z. Cheng P. Qian C. Xu F. Yang Y. (2020). Rhein modulates host purine metabolism in intestine through gut microbiota and ameliorates experimental colitis. *Theranostics* 10 10665–10679. 10.7150/thno.43528 32929373 PMC7482825

[B75] Xu C. Guo R. Hou C. Ma M. Dong X. Ouyang C. (2023). Resveratrol regulates macrophage recruitment and M1 macrophage polarization and prevents corneal allograft rejection in rats. *Front. Med.* 10:1250914. 10.3389/fmed.2023.1250914 37937143 PMC10626464

[B76] Yamauchi K.-E. (2002). Review on chicken intestinal villus histological alterations related with intestinal function. *J. Poultry Sci.* 39 229–242. 10.2141/jpsa.39.229 39221135

[B77] Yang S. Xu W. Feng L. Zhang C. Yan C. Zhang J. (2022). Resveratrol improves the Digestive ability and the Intestinal Health of Siberian Sturgeon. *Int. J. Mol. Sci.* 23:11977. 10.3390/ijms231911977 36233280 PMC9569792

[B78] Yin W. Zhou Q.-L. Ouyang S.-X. Chen Y. Gong Y.-T. Liang Y.-M. (2019). Uric acid regulates NLRP3/IL-1β signaling pathway and further induces vascular endothelial cells injury in early CKD through ROS activation and K+ efflux. *BMC Nephrol.* 20:319. 10.1186/s12882-019-1506-8 31412804 PMC6694569

[B79] Yu D. Zhu W. Hang S. (2019). Effects of low-protein diet on the intestinal morphology, digestive enzyme activity, blood urea nitrogen, and gut microbiota and metabolites in weaned pigs. *Arch. Animal Nutr.* 73 287–305. 10.1080/1745039X.2019.1614849 31163993

[B80] Ząbek K. Wójcik R. Milewski S. Małaczewska J. Tański Z. Siwicki A. K. (2016). Effect of β-hydroxy-β-methylbutyrate acid on meat performance traits and selected indicators of humoral immunity in goats. *Jpn. J. Vet. Res.* 64 247–256. 10.14943/jjvr.64.4.247 29786174

[B81] Zhang J. He W. Yi D. Zhao D. Song Z. Hou Y. (2019). Regulation of protein synthesis in porcine mammary epithelial cells by L-valine. *Amino Acids* 51 717–726. 10.1007/s00726-019-02709-2 30798466

[B82] Zhang R. Zhang W. Bi Y. Tu Y. Ma T. Dong L. (2019). Sanguinarine and resveratrol affected rumen fermentation parameters and bacterial community in calves. *Animal Feed Sci. Technol.* 251 64–75. 10.1016/j.anifeedsci.2019.03.004

[B83] Zhang Y. Zhou L. Bao Y. L. Wu Y. Yu C. L. Huang Y. X. (2010). Butyrate induces cell apoptosis through activation of JNK MAP kinase pathway in human colon cancer RKO cells. *Chemico Biol. Interactions* 185 174–181. 10.1016/j.cbi.2010.03.035 20346929

[B84] Zhao M. Di L. Tang Z. Jiang W. Li C. (2019). Effect of tannins and cellulase on growth performance, nutrients digestibility, blood profiles, intestinal morphology and carcass characteristics in Hu sheep. *Asian Australasian J. Animal Sci.* 32 1540–1547. 10.5713/ajas.18.0901 31010984 PMC6718903

[B85] Zhao W. Huang X. Han X. Hu D. Hu X. Li Y. (2018). Resveratrol suppresses gut-derived NLRP3 inflammasome partly through stabilizing mast cells in a rat model. *Mediators Inflammation* 2018:6158671. 10.1155/2018/6158671 30670927 PMC6317093

[B86] Zheng C. Song B. Duan Y. Zhong Y. Yan Z. Zhang S. (2020). Dietary β-hydroxy-β-methylbutyrate improves intestinal function in weaned piglets after lipopolysaccharide challenge. *Nutrition* 78:110839. 10.1016/j.nut.2020.110839 32540677

[B87] Zheng J. Li B. Yan Y. Huang X. Zhang E. (2022). β-Hydroxy-β-methylbutyric acid promotes repair of sheep myoblast injury by inhibiting IL-17/NF-κB signaling. *Int. J. Mol. Sci.* 24:444. 10.3390/ijms24010444 36613892 PMC9820147

[B88] Zhu K. Zhang Y. Zhang F. Wu Z. Su Q. Hou S. (2024). The Effects of dietary resveratrol and β-Hydroxy-β-Methylbutyric acid supplementation at two protein levels on the ruminal microbiome and metabolome of tibetan sheep. *Agriculture* 14:936. 10.3390/agriculture14060936

[B89] Zhu X. Liu Q. Wang M. Liang M. Yang X. Xu X. (2011). Activation of Sirt1 by resveratrol inhibits TNF-α induced inflammation in fibroblasts. *PLoS One* 6:e27081. 10.1371/journal.pone.0027081 22069489 PMC3206084

