# Supplementary material for: Exploring the effects of resveratrol and β-hydroxy-β-methylbutyric acid under different protein levels on the ileal health of tibetan sheep
Source: Front Microbiol. 2025 Jul 21;16:1612170. doi: 10.3389/fmicb.2025.1612170 (PMC12319562; doi:10.3389/fmicb.2025.1612170)
Supplement: Supplementary file 1 [file Table_1.docx]

Table S1. Composition and nutrient levels of feed ingredients.

| Ingredients | Chemical composition | Content (%) |
| --- | --- | --- |
| Corn | Crude protein (%) | 7.03 |
|  | Ether extract (%) | 2.90 |
|  | Neutral detergent fiber (%) | 9.90 |
|  | Acid detergent fiber (%) | 3.10 |
|  | Crude fiber (%) | 24.60 |
| Soybean meal | Crude protein (%) | 43.90 |
|  | Ether extract (%) | 9.70 |
|  | Neutral detergent fiber (%) | 13.60 |
|  | Acid detergent fiber (%) | 9.60 |
|  | Crude fiber (%) | 6.34 |
| Rapeseed meal | Crude protein (%) | 31.40 |
|  | Ether extract (%) | 12.80 |
|  | Neutral detergent fiber (%) | 20.70 |
|  | Acid detergent fiber (%) | 16.80 |
|  | Crude fiber (%) | 20.47 |
| Cottonseed meal | Crude protein (%) | 16.03 |
|  | Ether extract (%) | 9.00 |
|  | Neutral detergent fiber (%) | 32.10 |
|  | Acid detergent fiber (%) | 22.90 |
|  | Crude fiber (%) | 22.07 |
| Palm meal | Crude protein (%) | 16.03 |
|  | Ether extract (%) | 5.08 |
|  | Neutral detergent fiber (%) | 59.81 |
|  | Acid detergent fiber (%) | 35.26 |
|  | Crude fiber (%) | 15.85 |
